# Reply to Mizera and Strunga: Evidence for the Bolaven impact crater and its ejecta

**DOI:** 10.1073/pnas.2401580121

**Published:** 2024-05-13

**Authors:** Kerry Sieh, Jason Herrin, Dayana Schonwalder Angel

**Affiliations:** ^a^Earth Observatory of Singapore, Nanyang Technological University, Singapore 639798, Singapore; ^b^Asian School of the Environment, Nanyang Technological University, Singapore 639798, Singapore; ^c^Department of Geosciences, National Taiwan University, Taipei 10617, Taiwan; ^d^Institute of Earth Sciences, Academia Sinica, Taipei 11529, Taiwan; ^e^Facility for Analysis Characterisation Testing Simulation, Nanyang Technological University, Singapore 639798, Singapore; ^f^Escuela de Geología, Universidad Industrial de Santander, Bucaramanga, Santander CP 680002, Colombia

Five independent, multidisciplinary, field-based lines of evidence indicate that the source crater of the Australasian strewn field lies buried within a basaltic field in southern Laos (1, 2). In this short-format rebuttal to two recent critics (3, 4), we address just two of their several contentions.

**Fig. 1.**
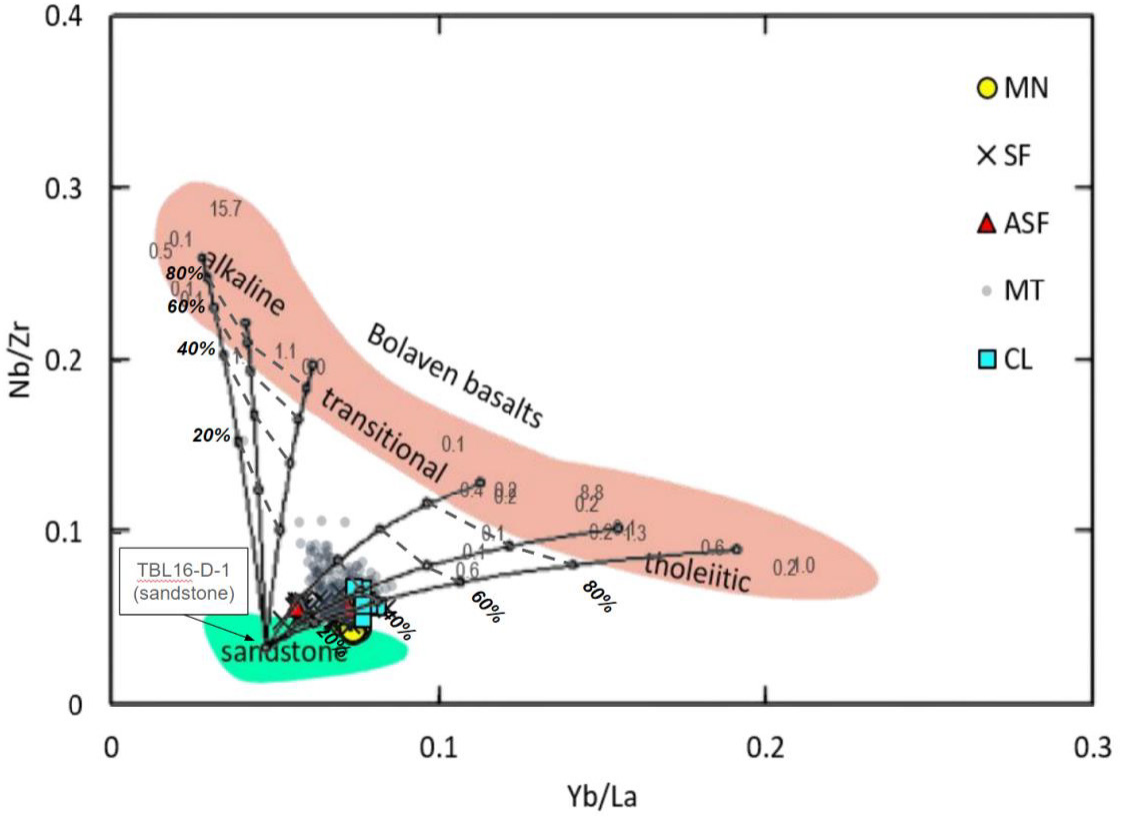
Binary mixing models overlain onto ref. 4’s figure 2, which is based on our (2, figure S5), illustrate addition of six basalts of varying composition (ranging from alkaline to tholeiitic) to a typical Bolaven sandstone (our sample TBL16-D-1). Percentages of basalt addition appear along the mixing lines. See ref. 4, figure 2 for description of symbols.

They argue that trace-element systematics preclude a significant basaltic component in Australasian tektites. To the contrary, our Fig. 1 overlay of binary mixing models onto their figure 2 demonstrates that tektite compositions are consistent with a 30 to 40% basaltic component, in agreement with estimates from our principal component analysis of major elements [(1) p. 3 and figure S5].

Abundant stratigraphic and sedimentological evidence favors formation and emplacement of the regionally extensive Bolaven diamicton by impact-related processes. It is normally graded, contains unabraded tektites in its upper portions, and thickens and coarsens toward the plateau, all of which are consistent with theory and experiment [(5) and (2), figure 12]. Its fine component bears chemical residues of basalt even at some sites in drainages without basaltic outcrops. At some sites very near the impact site, diamicton clasts have basaltic affinities even though the bed rests upon sandstone or mudstone bedrock [(2) p. 5, figures 6B and S12)]. The argument for postdepositional basaltic contamination of these deposits, which calls upon unspecified effects of “local geomorphology and hydrology” “within a lake area” (4), is untenable. It ignores the character of the sediments, the position of some of the deposits on high, flat, erosional surfaces without upslope basaltic sources, and that the lakes are postimpact modern reservoirs for hydroelectric power generation (6) that lie in canyons cut into Mesozoic fluvial sandstones (7).

Their arguments that we have misinterpreted the bouldery diamicton elsewhere confuse and conflate two distinctly different types of outcrop. One type is the complex diamiction depicted in the composite sketch of our figure 2 and illustrated with many examples. These we explain as deposits of the ejecta curtain. The other type is represented by three fields of dislodged but not ejected sandstone boulders just outside the perimeter of the buried crater [(1) p. 7, figure S16, and (2) p. 9, figures S2 and S7]. We propose the latter to have been lifted from subjacent bedrock by shock-related processes earlier in the impact process [(2) p. 9].

Incidentally, other recent field-based studies reinforce the Bolaven-impact hypothesis (8–10). Although some argue that definitive proof of the Bolaven impact crater will depend on drilling into it (11), we contend that discovery of the Bolaven diamicton essentially puts the case to rest and should serve to encourage further studies of the environmental effects of the impact and the detailed nature of its crater.
